# Supplementary material for: Folliculin regulates mTORC1/2 and WNT pathways in early human pluripotency
Source: Nat Commun. 2019 Feb 7;10:632. doi: 10.1038/s41467-018-08020-0 (PMC6367455; doi:10.1038/s41467-018-08020-0)
Supplement: Supplementary file 1 — Supplementary Information [file 41467_2018_8020_MOESM1_ESM.pdf]

Supplementary information

Folliculin regulates mTORC1/2 and WNT pathways in early human pluripotency

Mathieu et al.

SUPPLEMENTARY FIGURES

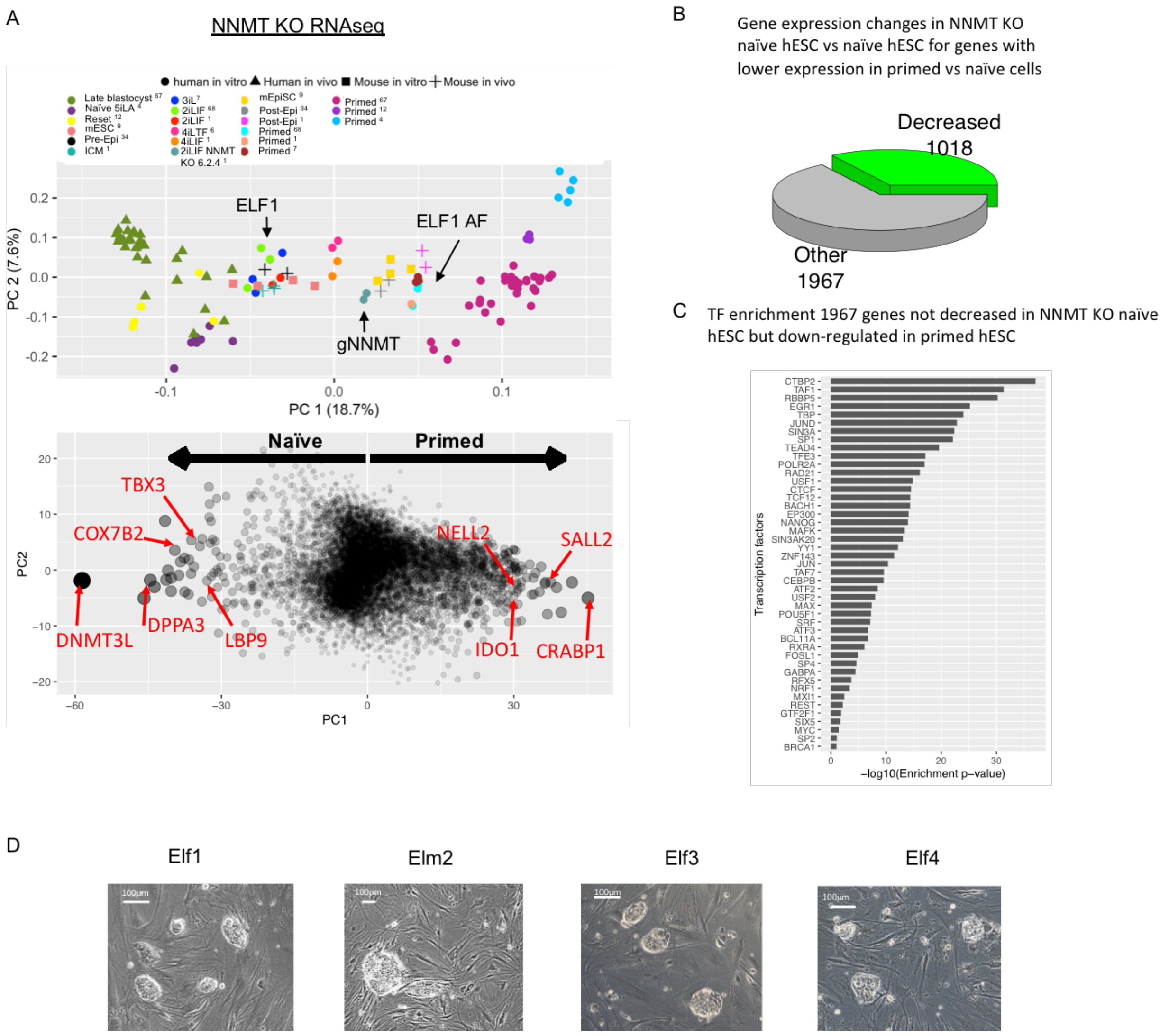

Supplementary Figure 1. *NNMT* KO partially move naïve hESC toward primed state
A. PCA plot of RNA-seq samples from human and mouse embryo as well as naïve and primed hESC after batch effects correction (top) and genes contributing to naïve vs. primed separation (bottom). *NNMT* CRISPR KO sample in 2iL-I-F moved toward primed state. Known marker genes are labeled. B. 66% (1967) genes with lower expression level in primed vs. naïve hESC did not show any decreased expression in *NNMT* CRISPR KO, indicating that they do not fully transition to primed state. C. Enrichment of transcription factor targets in these 1967 genes that are not downregulated by *NNMT* KO. D. Bright field images of newly derived naïve hESC Elf1, Elm2, Elf3 and Elf4 cultured in 2iL-I-F media.

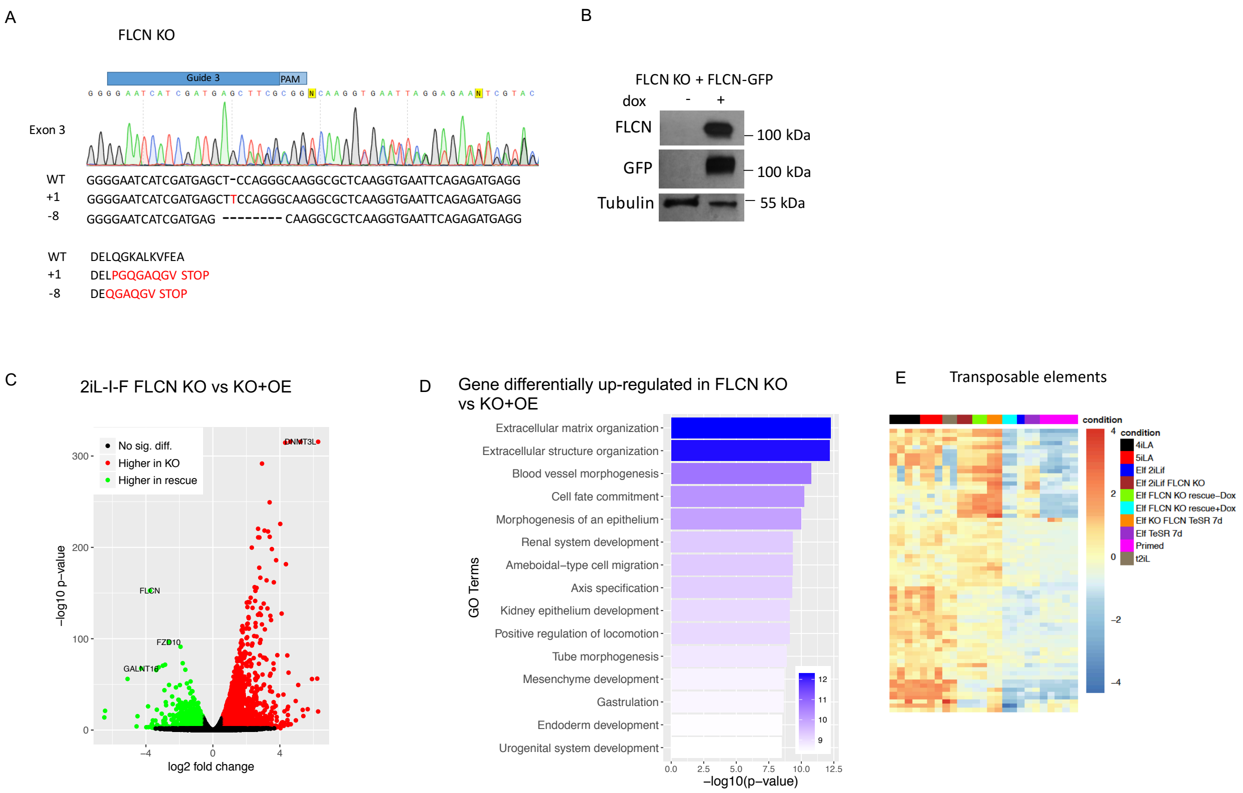

Supplementary Figure 2. Generation of FLCN KO and FLCN OE hESC

A. Trace file from Sanger sequencing of FLCN mutations in Elf1 2iL-I-F *FLCN* KO. B. Overexpression of FLCN-GFP fusion protein analyzed by Western blot. C. Volcano plot representation of differential expression analysis in Elf1 2iL-I-F FLCN KO vs Elf1 2iL-I-F FLCN KO+FLCN OE. D. Gene ontology analysis of genes differentially up-regulated in Elf1 2iL-I-F FLCN KO vs Elf1 2iL-I-F FLCN KO+FLCN OE. E. A subset of early naïve transposable elements [4,12] is up-regulated in naïve Elf1 FLCN KO compared to WT naïve Elf1. Values represent log2 fold change from the mean for each TRE.

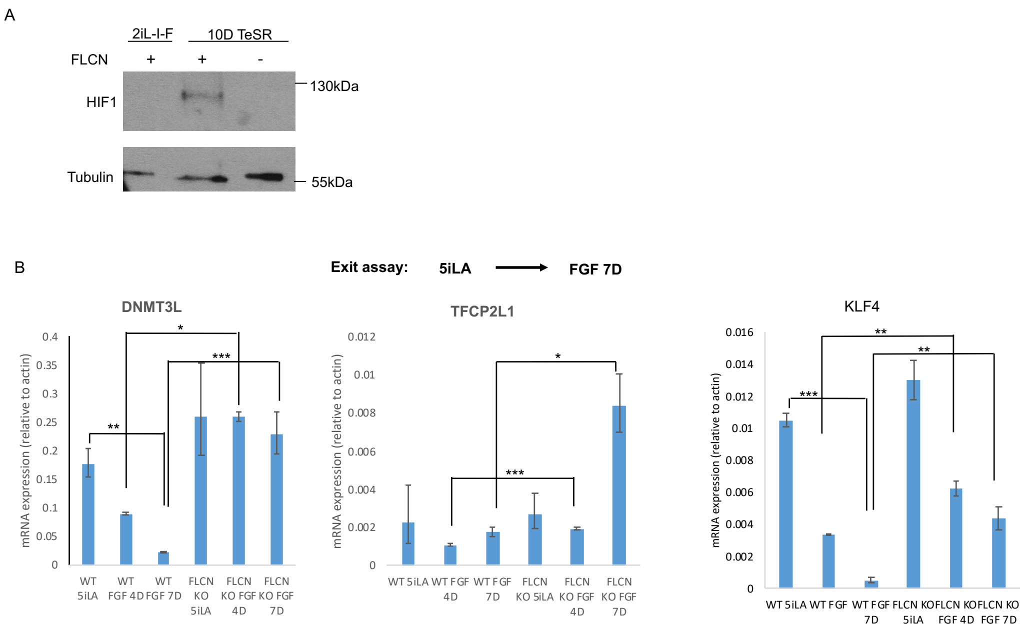

Supplementary Figure 3. FLCN controls the exit from naïve pluripotency
A. Western blot analysis of primed marker HIF1α in naïve 2iL-I-F hESC and 10D TeSR hESC WT and *FLCN* KO. B. qPCR analysis of naïve markers DNMT3L, TFCP2L1 and KLF4 upon exit from naïve state (5iLA to 7-day FGF). S.e.m.; *p<0.05, **p<0.005, ***p<0.001; 2-tailed t-test, n=3 to 6 biological replicates.

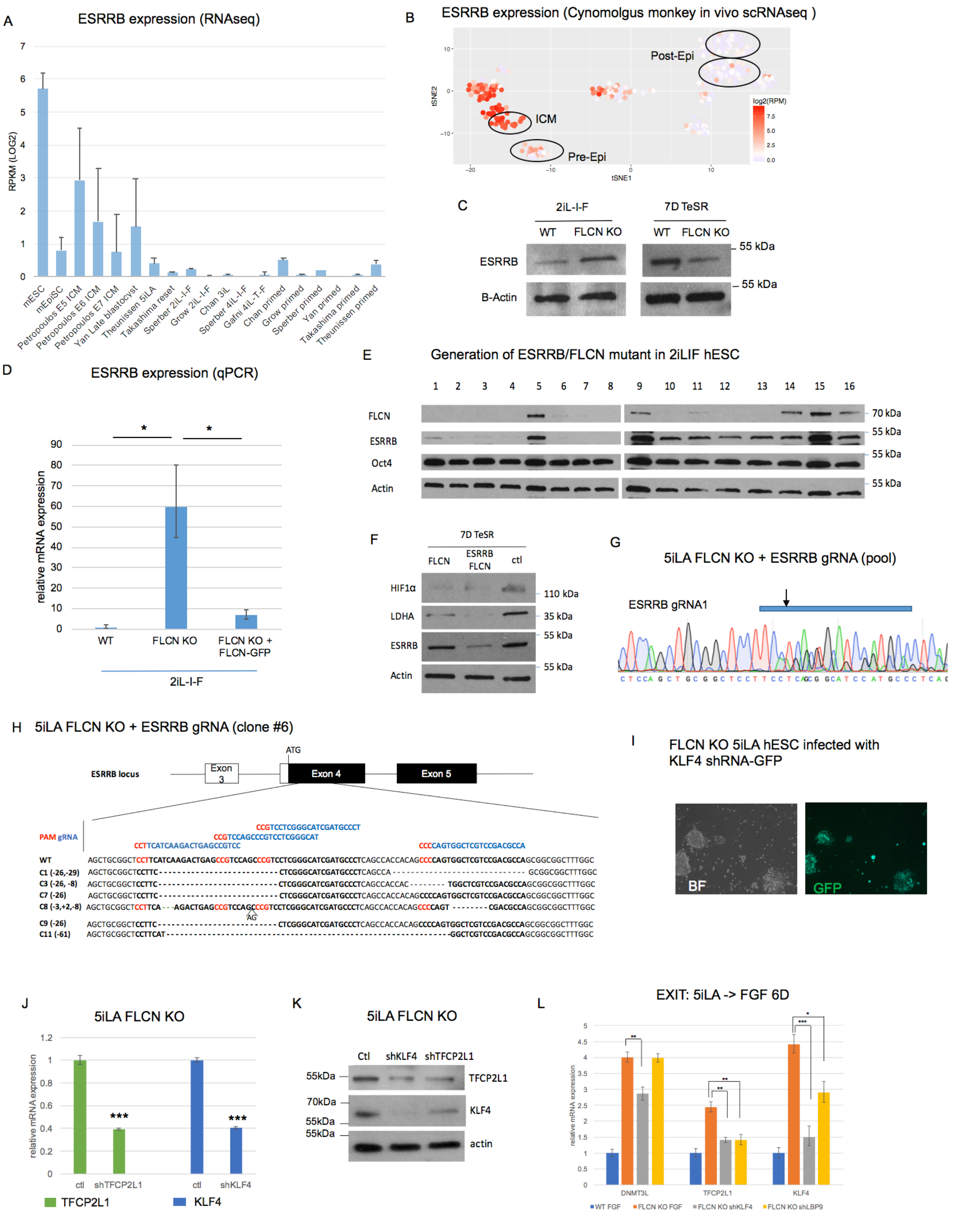

Supplementary Figure 4. ESRRB, KLF4 and TFCP2L1 functions in FLCN KO hESC

A. ESRRB expression in human pre-implantation[67,76], naïve and primed hESC lines[1,6,7,11,12,68] and mESC lines[9]. B. tSNE analysis of ESRRB in *in vivo* blastocysts from non-human primate, cynomolgus monkey (Macaca fascicularis,[33]). C. ESRRB is up-regulated by *FLCN* KO in naïve hESC but not during the exit of naïve pluripotent state. Western blot analysis of ESRRB expression in Elf1 2iL-I-F and Elf1 7D TeSR in WT and *FLCN* KO. D. RT-qPCR analysis of ESRRB in naïve (Elf1 2iL-I-F) and during exit (Elf1 7-day TeSR) in WT and *FLCN* KO hESC. S.e.m.; *p<0.05, NS:non-significant; 2-tailed t-test, n=3 biological replicates. E. Western blot analysis of Elf1 2iL-I-F clones isolated after infection with FLCN and ESRRB gRNA. *ESRRB/FLCN* double mutants still express the pluripotent marker OCT4. F. *ESRRB/FLCN* double mutant does not rescue *FLCN* KO phenotype during the exit of naïve pluripotency. Western blot analysis of primed hESC markers (HIF1α, LDHA) in Elf1 7D TeSR, Elf1 7D TeSR *FLCN* KO and Elf1 7D TeSR *FLCN* KO/ESRRB knock-down mutant (clone 4 in Supplementary Figure 4E). G. Sanger sequencing trace file of the pool of WIBR3 5iLA cells infected with ESRRB sgRNA lentivirus. The area covered by the guide is showed in dark blue. H. Mutations in ESRRB gRNA region were sequenced from WIBR3 5iLA *FLCN* KO *ESRRB* KO hESC (clone 6 in Fig.4g). The expression of 4 gRNA targeting ESRRB from lentiCRISPR lentivirus resulted in premature STOP codons. I-K. Knock-down of KLF4 and TFCP2L1 by shRNA in WIBR3 *FLCN* KO. Expression of green fluorescence protein(GFP) one week after infection of WIBR3 5iLA *FLCN* KO with KLF4 shRNA-GFP lentivirus was assessed under a fluorescence microscope. BF:bright field (I). RT-qPCR (J) and Western blot (K) analysis reveal knock-down of KLF4 and TFCP2L1 at the RNA and protein level with shRNA against KLF4 and TFCP2L1, respectively. S.e.m.; ***p<0.001; 2-tailed t-test, n=3 technical replicates. L. RT-qPCR of naive hESC markers (TFCP2L1, KLF4 and DNMT3L) during the exit of naïve pluripotency in WIBR3 6D FGF, WIBR3 *FLCN* KO 6D FGF, WIBR3 *FLCN* KO/KLF4 KD and TFCP2L1 mutants 6D FGF. S.e.m.; *p<0.05, **p<0.01, ***p<0.001; 2-tailed t-test, n=3 to 6 biological replicates.

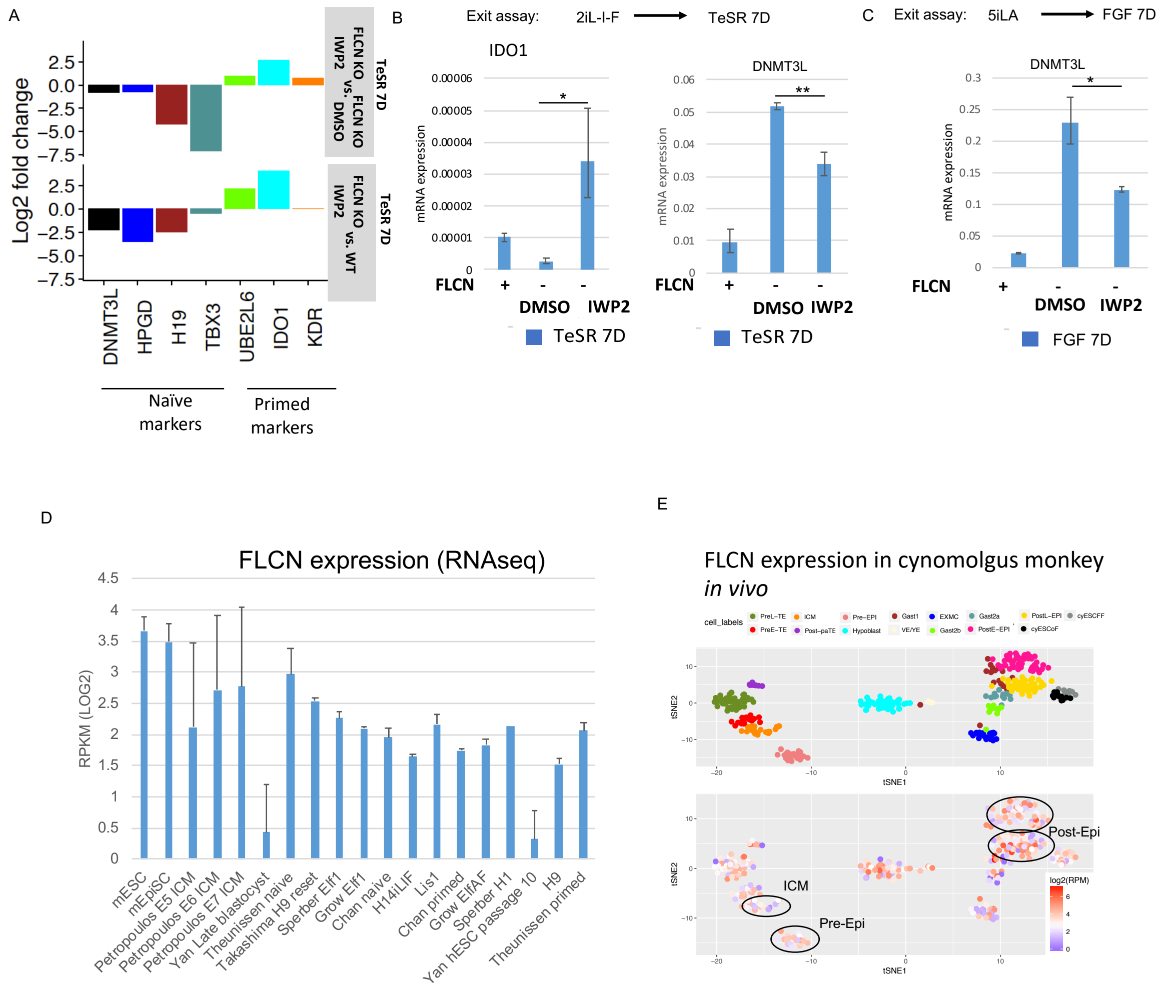

Supplementary Figure 5. WNT inhibitor rescues FLCN KO phenotype

A-C. Inhibition of WNT by IWP2 during exit of naïve state partially rescue FLCN KO phenotype as revealed by RNAseq (A) and RT-qPCR (B) analysis. Naïve markers are down-regulated and primed markers are up-regulated in Elf1 TeSR 7D *FLCN* KO IWP2 vs. in Elf1 TeSR 7D *FLCN* KO DMSO (A). Primed marker IDO1 is up-regulated and naïve marker DNMT3L down-regulated in *FLCN* KO cells treated with IWP2: Elf1 2iL-I-F exit (7D TeSR, B) and WIBR3 5iLA exit (7D FGF, C). S.e.m.; *p<0.05, **p<0.005; 2-tailed t-test, n=3 to 8 biological replicates. D. FLCN expression determined by RNA-seq in human pre-implantation[67,76], naïve and primed hESC lines[1,6,7,11,12,68] and mESC lines [9]. E. tSNE analysis of FLCN in *in vivo* blastocysts from non-human primate, cynomolgus monkey (Macaca fascicularis, [33]).

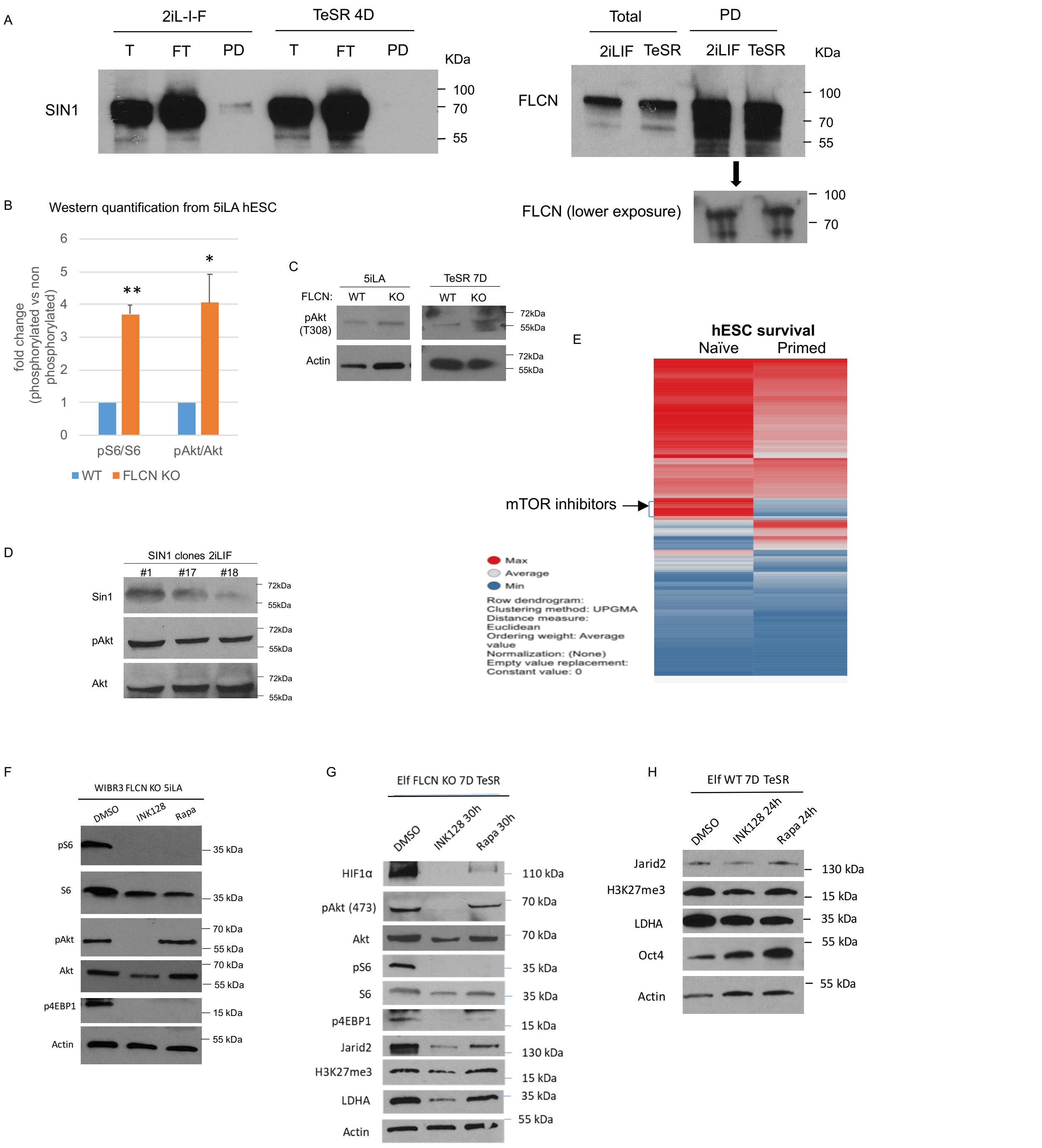

Supplementary Figure 6. mTOR regulation in hESC

A. Western analysis of SIN1 and FLCN after protein co-IP of FLCN-GFP in naïve (2iL-I-F) and TeSR (4D) hESC. T: total, FT: flow through, PD: pull down. A lower exposure of FLCN is shown in 2iL-I-F and TeSR PD. B.Quantification of phosphorylated versus non phosphorylated forms of AKT (p-Ser473) and S6 in wild type and *FLCN* KO WIBR3 5iLA. S.e.m.; *p<0.05, **p<0.005; 2-tailed t-test, n= 4 independent Western from biological replicates. C. Western blot analysis of pAKT(T308) in wild type or *FLCN* KO hESC cultured in naïve (5iLA) or primed (7D TeSR). conditions D. Western blot analysis of SIN1, pAKT(S473) and total AKT in SIN1 mutant clones cultured in naïve condition (2iLIF). E. Compound sensitivity profiles of naïve and primed hESC

were compared in a high throughput screen of 160 approved and investigational oncology drugs. F-H. Effect of mTORC1 inhibitor (Rapamycin) and mTORC1/2 inhibitor (INK128) on WT and *FLCN* KO hESC. WIBR3 *FLCN* KO 5iLA (F), Elf1 *FLCN* KO 7D TeSR (G) and Elf1 WT 7D TeSR (H) were treated for 24h or 30h with either DMSO (control), INK128 (100nM) or Rapamycin (100nM) (at day 6 of TeSR treatment for G and H).

SUPPLEMENTARY DATA

Supplementary Data 1. Elf1 2iL-I-F *NNMT* KO ChIPseq and RNAseq analysis
A. List of 913 genes showing increase H3K27me3 marks in primed hESC compared to 2iL-I-F naïve hESC but not showing any increase in 2iL-I-F *NNMT* KO (ChIP analysis, related to Fig.1b).
B. List of 1967 genes down-regulated in primed hESC (Elf1 AF) compared to naïve hESC (Elf1 2iL-I-F) but not down-regulated in Elf1 2iL-I-F *NNMT* KO (RNAseq analysis, related to Supplementary Fig.1B).

Supplementary Data 2. Results of CRISPR-Cas9 screen
For each CRISPR hit, the expression level of the corresponding gene in primed and naive hESC, as well as whether it is required for primed pluripotency[29] was listed.

Supplementary Data 3. RNAseq analysis of *FLCN* KO -/+ FLCN-GFP in 2iL-I-F
A. DESeq of naïve Elf1 2iL-I-F *FLCN* KO vs naïve Elf1 2iL-I-F *FLCN* KO + FLCN-GFP.
B. List of genes labelled in red in Fig.2e
C. List of transposable elements (TRE) presented in Supplementary Fig2E

Supplementary Data 4. RNAseq analysis of *FLCN* KO in 7D TeSR
A. DESeq of Elf1 TeSR 7D *FLCN* KO vs Elf1 TeSR 7D.
B. List of genes labelled in red in Fig.3c
C. List of genes labelled in green in Fig.3c
D. List of 673 TFE3 target genes up-regulated in *FLCN* KO 7D TeSR compared to WT 7D TeSR
E. DESeq of Elf1TeSR 7D *FLCN* KO IWP2 vs. Elf1TeSR 7D *FLCN* KO DMSO.

Supplementary Data 5. Proteomic analysis of FLCN-GFP pull down in Elf1 2iLIF and Elf1 3D TeSR (-/+ Dox 1µg/ml)

Supplementary Data 6. Sequences of gRNA and primers
A. Sequences of sgRNA
B. Sequences of shRNA
C. Sequences of PCR primers
D. Sequences of RT-qPCR primers

Supplementary Data 7. Statistics source data

SUPPLEMENTARY REFERENCE

76. Petropoulos, S. *et al.* Single-Cell RNA-Seq Reveals Lineage and X Chromosome Dynamics in Human Preimplantation Embryos. *Cell* **165**, 1012-1026 (2016).
