# Supplementary material for: Folliculin regulates mTORC1/2 and WNT pathways in early human pluripotency
Source: Nat Commun. 2019 Feb 7;10:632. doi: 10.1038/s41467-018-08020-0 (PMC6367455; doi:10.1038/s41467-018-08020-0)
Supplement: Supplementary file 3 — Description of Additional Supplementary Files [file 41467_2018_8020_MOESM3_ESM.pdf]

Description of Additional Supplementary Files

Supplementary Data 1. Elf1 2iL-I-F NNMT KO ChIPseq and RNAseq analysis

A. List of 913 genes showing increase H3K27me3 marks in primed hESC compared to 2iL-I-F naïve hESC but not showing any increase in 2iL-I-F NNMT KO compared to naïve hESC (ChIP analysis).
B. List of 1967 genes down-regulated in primed hESC (Elf1 AF) compared to naïve hESC (Elf1 2iL-I-F) but not not down-regulated in Elf1 2iL-I-F NNMT KO compared to naïve hESC (RNAseq analysis).

Supplementary Data 2. Results of CRISPR-Cas9 screen

Supplementary Data 3. RNAseq analysis of FLCN KO -/+ FLCN-GFP in 2iL-I-F

A. DESeq of naïve Elf1 2iL-I-F FLCN KO vs naïve Elf1 2iL-I-F FLCN KO + FLCN-GFP.
B. List of genes labelled in red in Fig.2E
C. list of transposable elements (TE) presented in Supplementary Fig2F

Supplementary Data 4. RNAseq analysis of FLCN KO in 7D TeSR

A. DESeq of Elf1 TeSR 7D FLCN KO vs Elf1 TeSR 7D.
B. List of genes labelled in red in Fig.3C
C. List of genes labelled in green in Fig.3C
D. List of 673 TFE3 target genes up-regulated in FLCN KO 7D TeSR compared to WT 7D TeSR
E. DESeq of Elf1TeSR 7D FLCN KO IWP2 vs. Elf1TeSR 7D FLCN KO DMSO.

Supplementary Data 5. Proteomic analysis of FLCN-GFP pull down in Elf1 2iLIF and Elf1 3D TeSR (-/+ Dox 1µg/ml)

Supplementary Data 6. Sequences of gRNA and primers

A. Sequences of sgRNA
B. Sequences of shRNA
C. Sequences of PCR primers
D. Sequences of RT-qPCR primers

Supplementary Data 7. Statistics source data
